# Paired-agent imaging as a rapid *en face* margin screening method in Mohs micrographic surgery

**DOI:** 10.3389/fonc.2023.1196517

**Published:** 2023-06-22

**Authors:** Veronica C. Torres, Sassan Hodge, Joshua J. Levy, Louis J. Vaickus, Eunice Y. Chen, Matthew LeBouef, Kimberley S. Samkoe

**Affiliations:** ^1^ Thayer School of Engineering, Dartmouth College, Hanover, NH, United States; ^2^ Geisel School of Medicine, Dartmouth College, Hanover, NH, United States; ^3^ Department of Pathology and Laboratory Medicine, Dartmouth Hitchcock Medical Center, Lebanon, NH, United States; ^4^ Department of Dermatology, Dartmouth Hitchcock Medical Center, Lebanon, NH, United States; ^5^ Quantitative Biomedical Sciences, Geisel School of Medicine, Dartmouth College, Hanover, NH, United States; ^6^ Department of Epidemiology, Geisel School of Medicine, Dartmouth College, Hanover, NH, United States; ^7^ Department of Surgery, Dartmouth Hitchcock Medical Center, Lebanon, NH, United States

**Keywords:** Mohs micrographic surgery, paired-agent imaging, surgical margins, fluorescence, image-guided pathology, epidermal growth factor receptor, skin cancer

## Abstract

**Background:**

Mohs micrographic surgery is a procedure used for non-melanoma skin cancers that has 97-99% cure rates largely owing to 100% margin analysis enabled by *en face* sectioning with real-time, iterative histologic assessment. However, the technique is limited to small and aggressive tumors in high-risk areas because the histopathological preparation and assessment is very time intensive. To address this, paired-agent imaging (PAI) can be used to rapidly screen excised specimens and identify tumor positive margins for guided and more efficient microscopic evaluation.

**Methods:**

A mouse xenograft model of human squamous cell carcinoma (*n* = 8 mice, 13 tumors) underwent PAI. Targeted (ABY-029, anti-epidermal growth factor receptor (EGFR) affibody molecule) and untargeted (IRDye 680LT carboxylate) imaging agents were simultaneously injected 3-4 h prior to surgical tumor resection. Fluorescence imaging was performed on main, unprocessed excised specimens and *en face* margins (tissue sections tangential to the deep margin surface). Binding potential (BP) – a quantity proportional to receptor concentration – and targeted fluorescence signal were measured for each, and respective mean and maximum values were analyzed to compare diagnostic ability and contrast. The BP and targeted fluorescence of the main specimen and margin samples were also correlated with EGFR immunohistochemistry (IHC).

**Results:**

PAI consistently outperformed targeted fluorescence alone in terms of diagnostic ability and contrast-to-variance ratio (CVR). Mean and maximum measures of BP resulted in 100% accuracy, while mean and maximum targeted fluorescence signal offered 97% and 98% accuracy, respectively. Moreover, maximum BP had the greatest average CVR for both main specimen and margin samples (average 1.7 ± 0.4 times improvement over other measures). Fresh tissue margin imaging improved similarity with EGFR IHC volume estimates compared to main specimen imaging in line profile analysis; and margin BP specifically had the strongest concordance (average 3.6 ± 2.2 times improvement over other measures).

**Conclusions:**

PAI was able to reliably distinguish tumor from normal tissue in fresh *en face* margin samples using the single metric of maximum BP. This demonstrated the potential for PAI to act as a highly sensitive screening tool to eliminate the extra time wasted on real-time pathological assessment of low-risk margins.

## 1 Introduction

**Figure 1:**
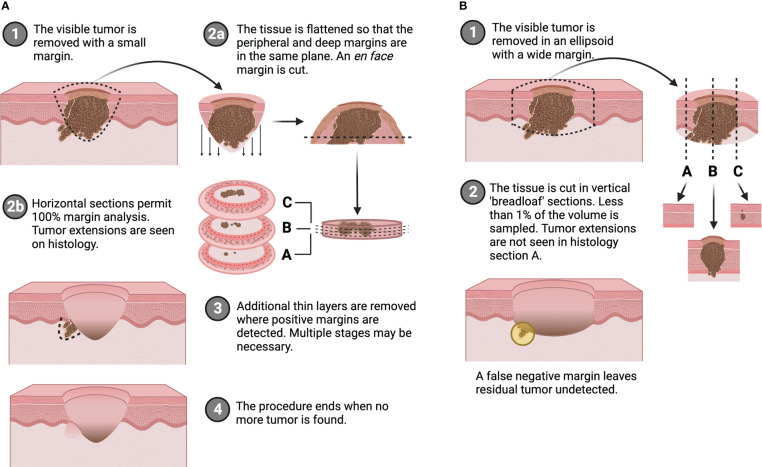
Comparison of **(A)** *en face* margin analysis used in Mohs micrographic surgery and **(B)** traditional wide local excision with breadloaf histology. Created with BioRender.com.

The presence or absence of tumor at the edges of a surgical resection specimen has significant prognostic implications. A known positive margin requires additional surgery and/or adjuvant therapy. An unknown or false negative margin increases the likelihood of tumor recurrence, which results in worsening morbidity and quality of life as well as decreased survival, placing a significant burden on patients and healthcare systems (1). Despite this importance of negative margins, standard methods of margin assessment do not sufficiently evaluate the whole excised sample. During conventional wide local excision (WLE), a 5-20 mm cuff of normal tissue is removed with the lesion, the specimen is permanently fixed, cut in vertical “breadloaf” sections, stained with hematoxylin and eosin (H&E), and then analyzed by a pathologist. While this lengthy and laborious procedure can take 1-7 days, less than 1% of resection margins are histologically evaluated (2). The outcome is a positive margin rate of 8.85% on average for the ten most common solid tumors (range: 4.32-35%) (1). As a result of incomplete microscopic margin analysis, patients are incorrectly diagnosed as tumor-free (i.e., with unidentified positive margins or false negative margins) and are not offered potentially life-saving treatment such as additional surgery or chemo/radio/immunotherapy. Alternatively, Mohs Micrographic Surgery (MMS) is an iterative approach for margin assessment that uses intraoperative frozen, tangential or *en face* sections to permit microscopic interrogation of 100% of surgical margins (**Figure 1**). As a result, high risk non-melanoma skin cancers (NMSC) that employ MMS, boast cure rates as high as 99% and recurrence rates ≤ 4% (3–5). The downside, however, is that MMS can be limited by the size of the resection specimen as tissue processing and histologic analysis times increase dramatically with tumor size (6). Moreover, only about 33.5% of slides read actually contain positive margins, meaning the majority of time is spent analyzing low-risk tissue (7).

Various imaging modalities have been investigated to address the problem of inadequate real-time margin evaluation, but none has shown overwhelming evidence in diagnostic ability and feasibility to compete with conventional methods. Nonetheless, fluorescence has been indicated as one of the most promising image methodologies for margin assessment, where single-agent imaging (SAI) – application of an exogenous imaging agent – can be used to enhance contrast (8). Most notably, fluorescence imaging has the advantage that it can be performed on whole, fresh tissues, thereby eliminating the lengthy histopathological steps of fixing, sectioning, staining and slide reading. This promise was demonstrated in clinical work reported by van Keulen et al. where they used a fluorescently labeled antibody to correlate signal intensity with margin distance, and therefore identify the closest margin on the deep surface of head and neck cancer specimens in real-time (9). In addition, Steinkamp et al. were able to distinguish positive surgical margins in clinical squamous cell carcinoma (SCC) samples based on tumor-to-background ratios using a pH-activatable fluorescent probe (10). The limitation of fluorescence imaging, however, is that sensitivity is depth limited. The above studies overcame this by imaging the deep margin of tumor specimens, such that detection sensitivity was greatest on the surface of interest. While *ex vivo* detectability was not hampered by depth penetration in these works, a common finding that can affect contrast and accuracy was non-specific signal.

An alternative to maintain histological specificity but still image whole tissues, is optical sectioning. This method enables the same microscopic resolution without the physical tissue processing by imaging focal planes throughout the depth of thick tissues. With respect to MMS in particular, techniques such as confocal mosaicking microscopy (CMM) (11, 12), microscopy with ultraviolet surface excitation (13), and two-photon fluorescence microscopy (14) or nonlinear microscopy (15) work to achieve this. All these efforts have shown success in achieving cellular resolution in fresh tissues, and demonstrated high sensitivity and specificity, as well as strong concordance with conventional histology. Fluorescence CMM was even tested in a clinical MMS setting and produced 88% sensitivity and 99% specificity in detecting positive basal cell carcinoma (BCC) margins (12). However, regardless of such successes, wide-spread adoption of these techniques remains elusive because of costly optics for high resolution scanning, the requirement for specialized equipment to mount and flatten specimens (necessary for mosaicking), and the ongoing challenge of non-specific staining that reduces contrast (11–15). Moreover, optical sectioning still requires interpretation from a pathologist or surgeon, with the added learning curve of reading virtually colored and stitched samples. Alternatively, machine learning algorithms can be used to rapidly read whole slide images, but successful application relies heavily on the quality of the images. Frozen and optical sections (produced from the techniques mentioned above) alike, suffer from routine factors such as blur, tissue tears and folds, color inconsistencies, and mosaicking artifacts (16–19). Therefore, there is a need for a more efficient methodology to assess margins without compromising the specificity and resolution of traditional histology.

To address this, an approach is proposed that combines the rapid imaging capabilities of fluorescence and the comprehensive evaluation of *en face* margins used in MMS. Fluorescence paired-agent imaging (PAI) is a well-validated technique that offers true quantification of receptor binding and overcomes non-specific signal by employing simultaneous administration of targeted and non-targeted imaging agents (20–22). Furthermore, with MMS techniques, removal of the bulk portion of the tumor offers greater contrast of the tissue region of interest (i.e., the margin), and still permits total analysis as the peripheral and deep margins are flattened onto the same plane (**Figure 1A**). By considering only the *en face* margin (as opposed to the whole excised specimen conventionally used in fluorescence imaging), confounding signal from depth limitations inherent to optical imaging can be reduced. Leveraging these specific and sensitive detection capabilities (23, 24), it was hypothesized that PAI of *en face* tissue margins can be used as a rapid screening tool to identify suspicious regions, and thereby streamline the samples requiring time-intensive histological attention. Fluorescence PAI can overcome the rate-limiting steps of histology and pathological interpretation, and margin imaging addresses the challenge of optical depth sensitivity. To demonstrate this, the work presented here investigates a xenograft murine model of human SCC that underwent PAI and MMS-like tissue preparation. Diagnostic accuracy and contrast of PAI were explored to test its rapid screening capabilities, and main specimen (whole resected tissue) imaging was compared to *en face* margin imaging to examine the effects on image quality.

## 2 Methods

### 2.1 Imaging agents

The targeted and untargeted imaging agents used were ABY-029 and IRDye 680LT carboxylate, formed from the NHS ester as described previously, (LI-COR Biosciences, Lincoln, NE), respectively (24). ABY-029 is an anti-EGFR (epidermal growth factor receptor) Affibody molecule labeled with IRDye 800CW, which was synthesized at the University of Alabama Birmingham Vector Production Facility (Birmingham, AL) (25, 26).

### 2.2 Mouse xenograft model

Animal procedures were performed in accordance with protocols approved by the Institutional Animal Care and Use Committee (IACUC) at Dartmouth College. The cell line used in this study was FaDu – a human squamous cell carcinoma cell line that moderately expresses EGFR, and was purchased from ATCC (Manassas, VA). The cells were cultured according to ATCC specifications with the addition of 1% penicillin-streptomycin. Eight female, 6–8-week-old, athymic nude mice (Charles River Laboratories, Wilmington, MA) were implanted subcutaneously with 1x10^6^ FaDu cells in both hind legs. Tumors were left to grow to a size of ~1 cm^3^; those that grew into the skin rather than the muscle such that there was clear separation of tumor from surrounding tissue (i.e., no deep margin) were excluded from the study (*n* = 13 viable tumors). Six additional mice were used as controls, and three more served as the naïve group to account for non-specific signal and biological autofluorescence, respectively.

### 2.3 Fresh tissue fluorescence imaging

**Figure 2:**
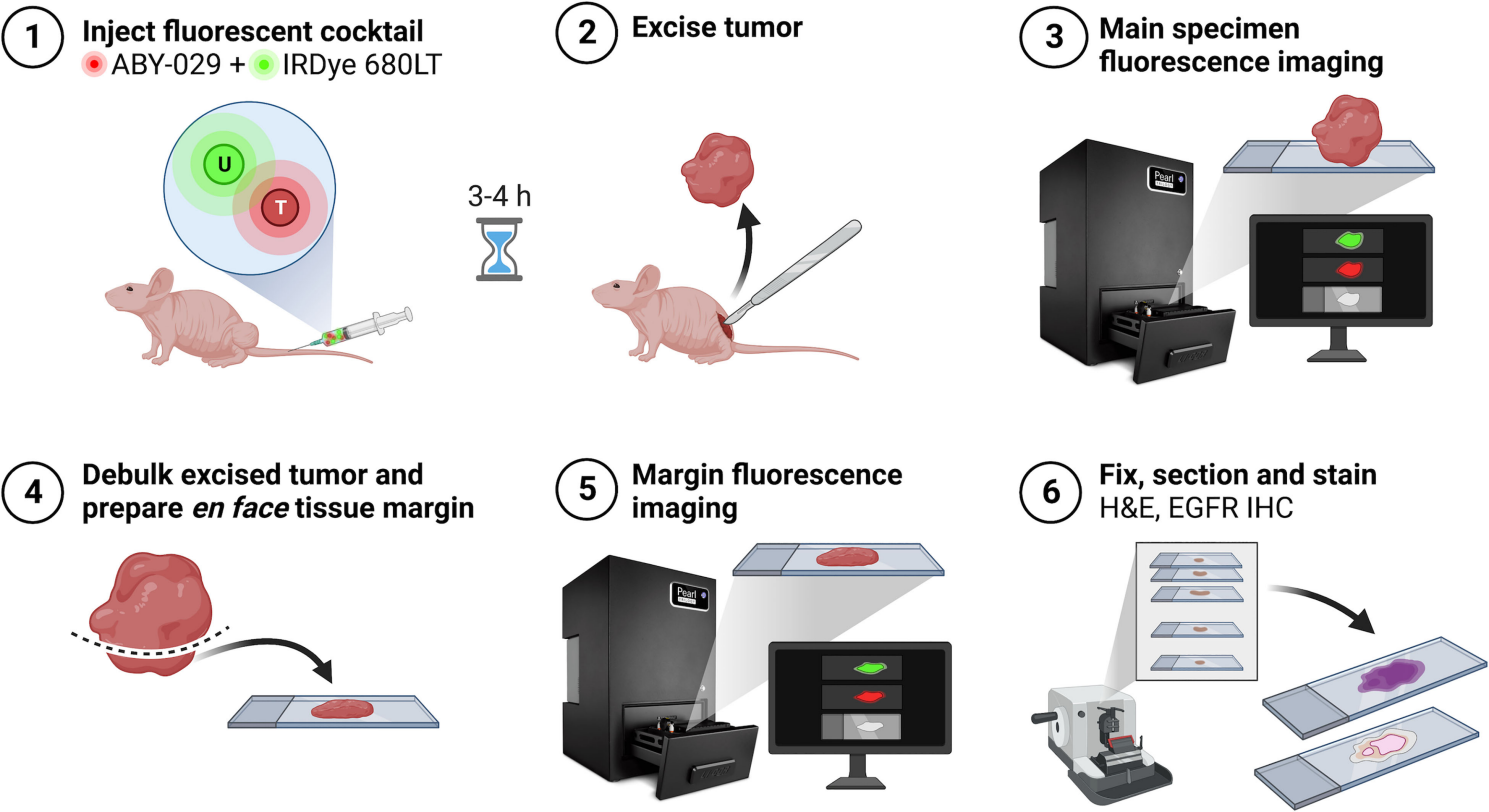
Experimental workflow schematic. Created with BioRender.com.

Prior to imaging, mice were injected via tail vein with 200 µL of the paired-agent solution (1:1 ABY-029:IRDye 680LT molar ratio at the mouse equivalent of a 180 nmol human dose of ABY-029) (27). Three to four hours post-agent administration, mice were sacrificed, and tumors were resected for “main specimen” imaging on the Pearl Impulse (LI-COR Biosciences). For PAI, images were collected in the white light, 700 and 800 nm channels. ABY-029 targeted fluorescence signal collected in the 800 nm channel was considered SAI. Next, the excised specimens were debulked and prepared by a Mohs surgeon (ML) to mimic an *en face* margin, i.e., the tissue was cut horizontally (tangent to the deep margin surface) such that the central portion of the tumor was removed and a 1-2 mm margin remained. Relaxing cuts were then made so that the peripheral margin could be flattened onto the same plane as the deep margin, and “margin” imaging on the Pearl was performed (**Figure 2**). An additional tissue layer was resected from the wound bed of one tumor that appeared invasive and was suspected to have a positive deep margin. This was handled and imaged similarly to a “margin” tissue.

Control mice underwent the same paired-agent procedure; however, skin and muscle tissue were acquired as they were non-tumor bearing. These served to monitor relative normal tissue agent uptake. Naïve mice had no tumors and were not administered any imaging agents in order to observe endogenous levels of autofluorescence.

### 2.4 Pathology

Following imaging, the fresh tissues were placed in 10% buffered formalin (Biochemical Sciences Inc, Swedesboro, NJ) in histological cassettes for fixation. All tissue handling and histopathological staining were performed by the institutional Pathology Translational Research Program shared resource (Geisel School of Medicine at Dartmouth, Dartmouth Hitchcock Medical Center and Dartmouth Cancer Center). Ten, 4-µm thick sections were cut for each tissue – the first five in 100 µm steps, and the other five in 200 µm steps. Because of its size, one tumor was sectioned in 200 µm intervals throughout the 10 sections, and the additional deep margin resected was sectioned every 50 µm for the extent of the tissue. For every section, two consecutive slices were taken – one for standard H&E and the other for EGFR immunohistochemistry (IHC) staining. All tissue slices were scanned on the Odyssey M (LI-COR Biosciences) at 10 µm resolution. Images were saved as TIF files with 8-bit precision.

### 2.5 Fresh tissue image analysis

#### 2.5.1 Binding potential calculation

All image analysis was performed in MATLAB. Binding potential (BP) – a quantity directly proportional to available receptor concentration (28) – maps were produced by applying the following equation on a pixel-by-pixel basis:


(1) $$BP=\frac{I_{T}}{I_{U}CF}-1$$

where *I_T_* and *I_U_* are the targeted and untargeted agent fluorescence intensities observed in the 800 nm and 700 nm channels, respectively (28). The correction factor, *CF*, was calculated as


(2) $$CF=\frac{{\bar{I}}_{T,skin}}{{\bar{I}}_{U,skin}}$$

where ${\bar{I}}_{T,skin}$ and ${\bar{I}}_{U,skin}\;$ are the mean targeted and untargeted agent intensities, respectively, from a normal skin region. The correction factor was calculated independently for each sample (29).

#### 2.5.2 Image quantification

Mean and maximum signal of the entire tissues were measured for BP and targeted fluorescence. These were collected from BP maps and 800 nm channel images of ABY-029 signal, respectively. BP was evaluated because it is the quantitative measure of PAI and has been demonstrated to correlate with tumor burden (20–24, 28, 29). Contrast-to-variance ratio (CVR) was determined with the equation:


(3) $$CVR=\frac{S_{tumor\;}-\;S_{norm}}{\sqrt{\sigma_{tumor}^{2}\;+\;\sigma_{norm}^{2}}}$$

where *S_tumor_* and *S_norm_* are the signals (mean/maximum BP/targeted fluorescence) from tumor tissue (main, margin) and normal samples (control/naïve skin/muscle), respectively. The standard deviations for each are represented by $\sigma_{tumor}^{2}$ and $\sigma_{norm}^{2}$ . CVR was calculated for each metric against the various normal tissue types independently such that each had four measures. CVR was measured because it has been shown to be a superior metric of image quality and a good indicator of diagnostic ability as it is correlated with an “ideal observer” (30, 31). Receiver operating characteristic (ROC) curve analysis was carried out in MATLAB, and diagnostic parameters were determined: area under the curve (AUC), sensitivity, specificity, positive predictive value (PPV), negative predictive value (NPV), and accuracy.

### 2.6 Pathological image analysis

EGFR IHC-stained slices were aligned to produce volumetric estimates of the sectioned samples. Manual control point registration was performed between successive slices by applying a rigid geometric transformation with the built-in MATLAB function *imregtform*. This accounted for translation and rotation of the tissue during handling. Following this, color deconvolution using the Ruifrok and Johnston method (32, 33) was performed on individual aligned slices to isolate EGFR signal. The deconvolved red dye transmittance was converted to absorbance using *A* = log_10_(1/*T*), where *A* is absorption and *T* is transmission. Linear interpolation was employed between slices of the aligned stacks (raw IHC and isolated EGFR), and then summed through each pixel along the z-axis to produce single-image representations of the sample volume. These will be referred to as IHC-summed and EGFR-summed images.

### 2.7 Fresh tissue-pathology co-registration and image quality analysis

White light images of the fresh tissue, both main specimen and margin, were co-registered with corresponding IHC-summed images. Manual control point registration was employed again; however, similarity transformation matrices were used to account for changes in scale that may have occurred as a result of tissue shrinkage after fixation. The same transformations were applied to BP maps and targeted fluorescence images. Gaussian filtering with a 9x9 kernel was applied to EGFR-summed images to account for the difference in image resolution between pathology and fresh tissue scans (10 µm vs 85 µm, respectively).

Contrast and detection accuracy of the main specimen and margin imaging were assessed by comparing to the EGFR IHC stains, which were considered the gold standard in this study. Two high intensity regions were identified in the EGFR-summed images, and horizontal and vertical line profiles passing through these areas were analyzed. Since the BP and targeted fluorescence main and margin images were co-registered, the same line profiles were taken. A single line profile was made up of the mean of a 20-pixel row or column. Similarity between the red intensity curves from EGFR-summed images and BP/targeted fluorescence intensity curves were estimated as the Euclidean distance sum, i.e., the sum of the difference between corresponding points. A value closer to zero indicates greater similarity. The MATLAB function *pdist2* was used to calculate the Euclidean distance. The mean Euclidean distance sum of four line-profiles was reported for each imaging method (BP vs targeted fluorescence) and tumor tissue type (main vs margin). One sample was excluded from this analysis before performing any similarity evaluation because the fresh and fixed tissues could not be co-registered (there were significant differences in sample shape from tissue handling).

A parallel trapezium model formula was applied to the pathology images to estimate tissue volumes (34). Raw IHC aligned stacks were used to estimate the volume of whole samples, and aligned isolated EGFR stacks were used to estimate tumor volumes. With these values, a tumor percent volume was calculated. Pearson’s correlation coefficients were calculated between tumor percent volume and each imaging metric, method, and tissue type combination.

### 2.8 Statistical analysis

All statistical analyses were performed using Excel and MATLAB statistical toolboxes. Data in the text is presented as mean ± standard deviation, and box plots show the median and interquartile range. The BP and targeted fluorescence signal for each tumor group and normal tissue group, and CVR and mean Euclidean distance sum for each imaging method and metric were compared using a two-tailed t-test. Linear regression results are reported as Pearson’s correlation coefficient, r. Statistical significance was based on p<0.05.

## 3 Results

### 3.1 Fresh tissue tumor detection

**Figure 3:**
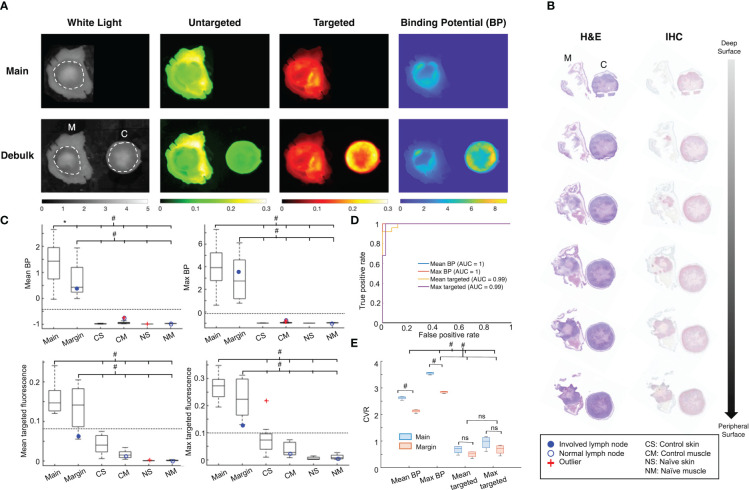
Fresh tissue paired-agent fluorescence imaging. **(A)** Representative main specimen and debulk tumor samples shown as the white light image, untargeted agent fluorescence image (IRDye 680LT signal in the 700 nm channel), targeted agent fluorescence image (ABY-029 signal in the 800 nm channel), and binding potential (BP) map. M: margin, C: central portion of tumor. Dashed white lines represent the skin-tumor boundary. **(B)** Corresponding histopathological staining of the debulked sample in **(A)** – standard hematoxylin and eosin (H&E) and epidermal growth factor receptor (EGFR) immunohistochemistry (IHC) – with horizontally cut serial sections. **(C)** Mean and maximum whole tissue BP and targeted fluorescence signal of tumor samples compared to normal skin and muscle tissue. Dotted lines indicate the optimal threshold for diagnostic accuracy determined from receiver operating characteristic (ROC) curve analysis. *p<0.05, ^#^ *p*<0.001, ns, not significant. **(D)** ROC curves for each imaging metric and method with corresponding area under the curves (AUCs). **(E)** Contrast-to-variance ratio (CVR) of each imaging metric and method.

Both PAI and single-agent imaging (SAI) with targeted fluorescence were able to detect all positive tumor margins. Representative images are shown in **Figure 3A**, where increased levels of signal were found in tumor regions for the targeted images and BP maps. Untargeted fluorescence, meanwhile, showed higher amounts of accumulation within skin. Signal localization was confirmed with histopathology (**Figure 3B**), and positive staining throughout the serial sections, with tumor growth moving from the deep to peripheral surface (~1.6 mm), demonstrated the depth sensitivity of the fluorescence imaging. To evaluate the ability of PAI and SAI to discriminate tumor from normal tissue using signal alone, whole tissue mean and maximum values of BP and ABY-029 fluorescence intensity were investigated (**Figure 3C**). For both imaging methods (PAI and SAI) and metrics (mean and max), main and margin tumor tissues were significantly different (*p*<0.001) from normal skin and muscle samples from control and naïve mice. As expected, average main specimen signal was greater than that of margin samples; however, the differences were not statistically significant except for the case of mean BP (*p*<0.05). Although PAI and SAI were both able to distinguish between tumor and normal tissue, PAI had a better diagnostic ability. Optimal thresholds were determined from ROC curve analysis (**Figure 3D**), which illustrated the advantage in tissue discrimination lent by PAI. Both mean and max BP showed clear separation of tumor tissues above, and normal tissue below the optimal threshold lines (NPV = 1, PPV = 1, sensitivity = 1, specificity = 1). Meanwhile, the optimal threshold for mean targeted fluorescence was within the range of tumor margin values, leading to a NPV of 0.95 and sensitivity of 0.92; and for max targeted fluorescence, the ideal cutoff intersected with control skin tissue values, resulting in a PPV of 0.96 and specificity of 0.98. All of this translated to 100% accuracy for mean and max BP, whereas the same measures for targeted fluorescence had reduced accuracies of 97% and 98%, respectively. While the relative diagnostic parameters were inferior for SAI, overall performance was still strong; therefore, contrast between the groups was evaluated to test for significant differences. CVR analysis revealed that BP consistently outperformed targeted fluorescence imaging for all tumor tissues (*p*<0.001) with an average CVR improvement of 4.4 ± 1.5 times (**Figure 3E**); and measures of maximum BP for main and margin samples offered the greatest contrast over other metrics (max BP CVR: 3.5 ± 0.04, 2.8 ± 0.04 for main and margin, respectively; mean BP: 2.6 ± 0.06, 2.1 ± 0.07; max targeted: 0.94 ± 0.26, 0.68 ± 0.18; mean targeted: 0.68 ± 0.16, 0.50 ± 0.12) – an average 1.7 ± 0.4 times improvement. BP for main tumor tissues also had significantly higher CVR than margin samples for both the mean and maximum values (*p*<0.001), whereas there was no significant difference when using targeted fluorescence.

### 3.2 Main specimen vs *en face* margin

**Figure 4:**
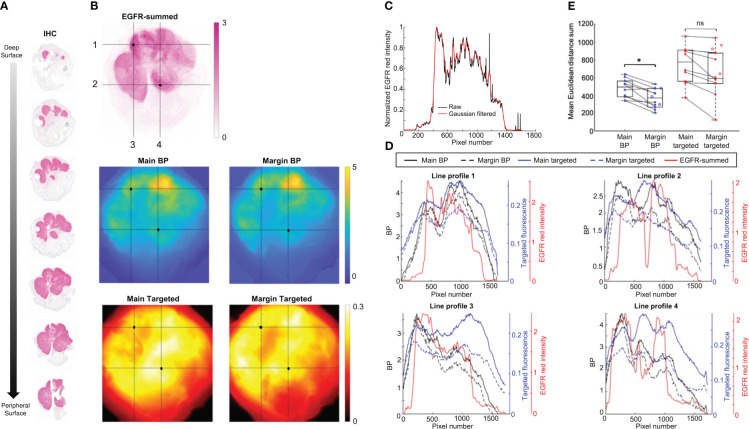
Representative fresh tissue and immunohistochemistry (IHC) comparison. **(A)** Horizontally cut serial sections with epidermal growth factor receptor (EGFR) IHC staining. **(B)** Co-registered isolated EGFR-summed image from serial sections in **(A)**, and main and margin binding potential (BP) and targeted fluorescence images. Horizontal and vertical line profiles labeled 1-4 were drawn over two high intensity regions (black dot) identified on the EGFR-summed image. **(C)** Representative line profiles from a raw EGFR-summed image, and after application of a Gaussian filter. **(D)** Line profiles of BP, targeted fluorescence, and filtered EGFR-summed red intensity for images from **(B)**. **(E)** Comparison of change in mean Euclidean distance sum from main specimen to margin imaging for BP and targeted fluorescence. Closed dots represent samples with matched pairs, while open dots are margin samples only. *p<0.05, ns, not significant.

Contrast and resolution of main and margin samples were assessed by comparing to IHC, which served as the benchmark for EGFR presence in this study. **Figure 4B** shows representative co-registered images of isolated and summed EGFR signal from an IHC stack (**Figure 4A**), as well as main and margin BP and targeted fluorescence. Visually, BP maps appeared to have better resolution than their single-agent counterparts, with patterning that more closely resembled the EGFR-summed image. Moreover, main tumor images – especially from targeted fluorescence alone – were blurrier than the margin images. To better demonstrate these observations, four line-profiles were plotted from each (**Figure 4D**). Red intensity curves were taken from EGFR-summed images that were Gaussian filtered to better match the collection resolution of the fluorescence acquisitions and to reduce noise. **Figure 4C** displays a sample plot before and after filtering – the overall trend of the signal was maintained while eliminating outliers. This was crucial for pair-wise comparison of line profiles from the various images. As illustrated in **Figure 4D**, main and targeted fluorescence line profiles were broader, with fewer and shallower peaks and troughs, which are indicative of poorer contrast and resolution. However, the margin BP plots were narrower and followed the fluctuations of the EGFR red intensity curves more consistently. This was quantified as the mean Euclidean distance sum for each sample, shown in **Figure 4E**. Margin BP had the lowest value (indicating the two observations were closer to one another) with a mean of 366 ± 115 and average improvement of 3.6 ± 2.2 times over other measures (main BP: 480 ± 110, main targeted: 749 ± 217, margin targeted: 641 ± 271). Margin imaging compared to main tumor imaging decreased the distance metric for both BP and targeted fluorescence, but there was only a statistically significant improvement when using PAI (*p*<0.05).

### 3.3 Fluorescence correlation with IHC

**Figure 5:**
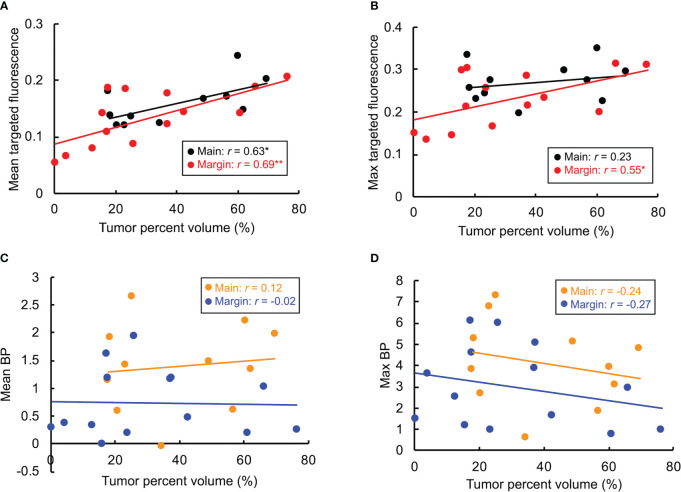
Correlation between tumor volume and targeted fluorescence or binding potential (BP) for main and margin specimens. Mean and maximum targeted signal and BP are shown in **(A-D)**, respectively. Linear regression results are reported as Pearson’s correlation coefficient, *r*. *p<0.05, **p<0.01.

As shown in **Figure 5**, linear regression results between tumor volume and signal revealed strong correlations with SAI for almost all cases, and either no- or weak-correlation when using BP (a weak correlation indicates signal is independent from sample size). Pearson coefficients for mean targeted fluorescence were 0.63 and 0.69 for main and margin samples, respectively, and 0.23 and 0.55 for max targeted intensity. Mean BP had main tumor and margin Pearson coefficients of 0.12 and -0.02, respectively, and -0.24 and -0.27 for maximum BP.

## 4 Discussion

Tumor free margins are critical for patient prognosis; yet the average positive surgical margin rate in the ten most common solid cancers in the United States is 8.85% (1) Positive surgical margins result in elevated healthcare costs due to repeat surgery or adjuvant therapy (1). False negative margins result from sampling error in standard histopathological evaluation with less than 1% of the margins examined microscopically (2). Several novel imaging techniques have been explored with the goal of intraoperative total margin analysis, however the same level of diagnostic reliability lent by the gold standard has not been met with equal feasibility (8). MMS used for NMSC is an existing technique that achieves this – *en face* sections containing 100% of surgical margins are snap-frozen and microscopically assessed in stages until tumor is no longer detected. Because this is an iterative procedure that still requires histological processing and assessment with such high scrutiny, the procedure is reserved for only small-sized tumors in high-risk areas (6). Fluorescence imaging has the ability to bypass lengthy histopathological procedures and assess whole, fresh samples, but lacks the necessary cellular resolution. The goal in this study was to demonstrate the ability of fluorescence PAI to rapidly screen resected samples to help guide MMS. The idea is not to change the technique of the established procedure; rather, to provide a method of stratification to eliminate unnecessary pathological work. This has important time- and cost-savings implications because >66% of evaluated slides are often negative (7), but all are equally investigated.

There were two salient findings in this study: first, that PAI could reliably detect tumor presence in fresh tissue samples with signal alone; and second, that imaging debulked margin tissue compared to the main specimen improved correspondence with EGFR IHC staining. The first result was noteworthy because it demonstrated the potential for PAI BP as a single metric to act as a rapid screening tool. This is especially useful for procedures like MMS where tissue size is a rate-limiting factor – larger samples are often grossed into halves or quadrants, resulting in more tissue blocks and subsequent slides that require embedding, sectioning, staining and histologic analysis. Thus, the ability to identify “definitely positive” or “definitely negative” samples could streamline the process by bypassing real-time pathology on a sample altogether. The data presented here suggests that PAI with maximum BP as the sole measure could achieve this with 100% accuracy and more reliably than the other metrics. This was emphasized for specific cases where tumor burden was low relative to tissue volume. For instance, one margin sample had a tumor percent volume of 0.4% but was correctly identified with a single whole tissue scan. It is likely that standard breadloaf histology would miss this, as<1% of total volumes are typically assessed with the method (2). MMS meanwhile, would have a better probability of detection; however, the pressure required of pathologists to identify such small lesions and the amount of time spent reading negative tissue is underscored here. An involved lymph node was also detected in the deep margin removed from the wound bed of another animal, which comprised 4% of the total tissue volume (range for all margin samples: 0.4-76%). As shown in **Figure 3C**, the calculated maximum BP of 3.6 was within a standard deviation of the mean (3.0 ± 1.9) and well above the optimal threshold (-0.05) delineating tumor from normal tissue, as determined by ROC analysis. Mean BP for the same tissue showed similar results, but with less discriminating separation (mean BP: 0.4, optimal threshold: -0.4). Moreover, maximum signal compared to the mean was superior (*p*<0.001) in increasing contrast (**Figure 3E**). Targeted fluorescence intensity signals were able to identify the positive margin, however with less reliability. The mean measure for the sample was below the optimal threshold (0.07 and 0.08, respectively), suggesting a false negative prediction; and while the maximum signal (max: 0.13, optimal threshold: 0.12) would be correctly classified, the value was the minimum of the range and outside one standard deviation of the mean (0.23 ± 0.07). In addition, lymph nodes in control and naïve mice were correctly classified as normal, confirming that BP and targeted signal were indeed from specific binding and not accumulation. These findings support the ability of PAI to quickly distinguish tumor from normal tissue in whole fresh specimens.

Linear regression showed weak or no correlation between BP and tumor volume (based on estimates of EGFR IHC) (**Figures 5C, D**). While it may seem counterintuitive, this was anticipated as BP is a measure of available receptor concentration. Since only one cell line was investigated here, the amount of EGFR remains the same; and as such, similar measures of BP among various samples would be expected. This further highlights the advantage that BP is not affected by volume. Conversely, **Figures 5A, B** show a strong correlation with targeted fluorescence and the amount of tumor present, with the exception of main specimen imaging measured with maximum signal (weak correlation, *r* = 0.23). The danger with this, similarly illustrated above, is that low tumor burden relative to whole tissue volumes are vulnerable to such low levels of signal that the possibility of a false negative increases. This also emphasizes the presence of non-specific signal when using only a single imaging agent (even a targeted one). Total targeted fluorescence signal is a contribution of both specifically bound agents and unbound uptake that increases with tissue size. PAI overcomes this by normalizing unwanted signal across the sample. The weak correlation for maximum targeted fluorescence in main specimens can be attributed to volume effects. The main sample is comprised of the core tumor and margin; thus, large amounts of signal come from the core, and because the sample is thick (>2 mm), much of the fluorescence is scattered giving the image a diffuse appearance. This has an averaging effect on the overall signal, and consequently, the maximum value remains similar despite the actual amount of tumor present. The relatively horizontal regression line in **Figure 5B** illustrates this.

The second notable result of this study was that imaging the debulked *en face* margin compared to the main specimen improved image quality, especially for PAI. Overall, the margin images had better contrast and resolution. Moreover, the similarity of BP images to IHC improved significantly, demonstrating the true binding and quantification of EGFR made possible with PAI. This is important for a couple of reasons. First, it further validates the ability of PAI to be used as a high-throughput screening technique – the gold standard diagnostic of MMS is pathology, and margin BP maps can provide images similar to IHC. Second, co-localization shows the potential for fluorescence guided-pathology. With better resolution, and thus localization, specific regions of the samples can be highlighted as more or less suspicious for tumor presence. This would allow pathologists to prioritize their analysis based on suspicious regions. The improvements provided by margin imaging can be explained by the effects of volume and limited depth penetration as described above. By removing the known and confounding signal of the tumor core, the margin (actual tissue of interest) could be assessed more reliably. It is acknowledged that H&E (not IHC) is the standard for MMS; however, IHC was assessed here so that a direct comparison could be made between BP/fluorescence signal and pathology. EGFR is the molecular target of ABY-029, and so the EGFR IHC staining served as a benchmark for the receptor’s presence and validation of actual binding. The purpose of H&E in this study was for diagnostic confirmation. Our previous work demonstrated strong diagnostic ability and correlation between PAI frozen sections and H&E (23), thus we expect similar agreement. Some loss in correlation however, can be anticipated when moving from frozen thin slices to fresh thick tissue.

While the results presented here proved promising, there were limitations to the study. The high diagnostic accuracy reported was likely influenced by the presence of only positive margins. This is in part owing to the cell line used and possibly the cell injection method. The FaDu tumors used here had a ball-like growth, which is not representative of the subclinical extensions often seen in high grade NMSCs treated by MMS. In addition, the tumors and mice themselves were small, leaving limited ability and room for more invasive growth, and the option to excise additional margin layers. Future studies will work to address these problems by exploring additional cell lines, adjusting the method of cell implantation (e.g., greater depth of injection, drag the needle to promote dispersed growth), and using rat or porcine animal models. It should also be noted that permanent sections were used here, not frozen as in MMS. The purpose of the pathology performed in this work was simply for validation; however, future studies will include frozen pathology for a more representative and direct comparison to standard methods. In addition, while the co-registration techniques employed were sufficient for a coarse assessment, the use of fiducials would aid in a more robust alignment such that pixel-wise analysis could be performed. This work served as a novel investigation of how PAI can be applied to improve total margin analysis in MMS. While that is the ultimate vision, this proof-of-concept study aimed to test the feasibility of the proposed solution. The goal was to use the current data as a training set to establish optimal metrics and thresholds for tissue discrimination, and as such, no validation set was included here. Future studies will apply the determined parameters for validation.

From a feasibility perspective, the PAI method proposed here has several benefits. The combination of ABY-029 and IRDye 680LT can offer stable contrast above normal tissue within 30 minutes of administration and for up to 5 h (24, 29). This would allow the current MMS practice, which can take up to several hours at most, to remain an outpatient procedure – an outcome favorable for patients and healthcare systems. Other imaging agents, meanwhile, require administration a day or more prior to surgery, meaning repeat visits to the hospital for patients (9, 10). In addition, only one application of paired-agents would likely be required for even longer, more complicated procedures with multiple layer removals because of the long duration of stable contrast. PAI is also beneficial because it can be integrated into the current MMS workflow without major disruption. The only change in the surgical suite (aside from pre-operative agent administration) would be the addition of back table imaging of the resected specimen – a process that requires only ~30 s. Importantly, PAI does not change the manner in which pathologists histologically assess the slides. An ongoing critique of optical sectioning microscopy methods developed for MMS is that there is a significant learning curve. Specific training is required to read digital mosaics and become accustomed to grayscale images if pseudo-coloring is not available (11–15). This is not a problem for PAI-guided pathology because the purpose of BP maps is simply to inform how many and/or in which order the slides are read. Additionally, we have demonstrated that PAI provides a signal that is interpretable across patient samples, unlike SAI where signal intensity can vary within positive samples due to variations in tumor physiology and optical properties (29).

PAI of *en face* margins provides a robust and rapid means of total margin analysis. It was demonstrated that a single metric of maximum BP could discriminate tumor from normal tissue with high diagnostic accuracy. These promising results support the potential for PAI to improve the efficiency of standard MMS thereby removing logistical hurdles of current workflows and pushing the limits of tissue sizes accepted for the procedure in various operative settings. This work has broad implications for additional tumor types where intraoperative margin status and tissue preservation is critical for patient outcomes including head and neck cancers.

## Data availability statement

The original contributions presented in the study are included in the article/supplementary material. Further inquiries can be directed to the corresponding author.

## Ethics statement

The animal study was reviewed and approved by Institutional Animal Care and Use Committee at Dartmouth College.

## Author contributions

KSS, ML, EYC and LJV conceptualized the study. KSS and VCT designed the study. VCT, SH and KSS conducted the experiments. EYC and ML performed the surgeries. VCT, SH and KSS analyzed the data. VCT drafted the manuscript. All authors contributed to the article and approved the submitted version.
